# Value of Measuring Anti-Carbamylated Protein Antibodies for Classification on Early Arthritis Patients

**DOI:** 10.1038/s41598-017-09657-5

**Published:** 2017-09-20

**Authors:** Cristina Regueiro, Laura Nuño, Ana M. Ortiz, Diana Peiteado, Alejandro Villalba, Dora Pascual-Salcedo, Ana Martínez-Feito, Isidoro González-Alvaro, Alejandro Balsa, Antonio González

**Affiliations:** 1 Experimental and Observational Rheumatology, Instituto de Investigacion Sanitaria - Hospital Clínico Universitario de Santiago, Santiago de Compostela, Spain; 2 Rheumatology Department, Instituto de Investigación Hospital Universitario La Paz (IDIPAZ), Madrid, Spain; 3 Rheumatology Department, Instituto de Investigación del Hospital de La Princesa (IIS-IP), Madrid, Spain; 4 Immuno-Rheumatology Department, Instituto de Investigación Hospital Universitario La Paz (IDIPAZ), Madrid, Spain

## Abstract

Classification of patients with rheumatoid arthritis (RA) as quickly as possible improves their prognosis. This reason motivates specially dedicated early arthritis (EA) clinics. Here, we have used 1062 EA patients with two years of follow-up to explore the value of anti-carbamylated protein (anti-CarP) antibodies, a new type of RA specific autoantibodies, for classification. Specifically, we aimed to determine whether the addition of anti-CarP antibodies to IgM rheumatoid factor (RF) and anti-cyclic citrullinated peptide (anti-CCP) antibodies, which are helpful in RA classification, improves it or not. Our analysis showed that incorporation of the anti-CarP antibodies to combinations of the other two antibodies (all joint by the OR Boolean operator) produces a modest increase in sensitivity (2.2% higher), at the cost of decreased specificity (8.1% lower). The cost-benefit ratio was more favorable in the patients lacking the other autoantibodies. However, it did not improve by considering different titer levels of the anti-CarP antibodies, or after exhaustively exploring other antibody combinations. Therefore, the place in RA classification of these antibodies is questionable in the context of current treatments and biomarkers. This conclusion does not exclude their potential value for stratifying patients in joint damage, disease activity, disability, or mortality categories.

## Introduction

Advances in the treatment of rheumatoid arthritis (RA) have shown that active drugs should be given as soon as possible^1, 2^. This idea has been framed in the concept of a window of opportunity for the best results, window that extends only for the first months since symptoms onset^3–6^. The benefits of treatment in this window include increased response rates, decreased disease activity, prevention of bone erosions, less disability, increased rates of remission, even of drug-free remission, and larger improvement in health quality scores^3–6^. These benefits are of large significance, but are demanding for the rheumatologist because often it is difficult to diagnose RA when the first symptoms appear. This task has been facilitated by the development of new RA classification criteria in 2010^2^. These criteria aim to define patients earlier in the disease course than the previous criteria from 1987^7^. This was accomplished by deleting some features associated with established RA, as rheumatoid nodules and radiographic changes, from the list of criteria, and by incorporating or increasing the weight of characteristics that are present early in the RA course. In this latter group of characteristics is remarkable the weight given to the RA autoantibodies, rheumatoid factor (RF) and anti-cyclic citrullinated peptide (anti-CCP). Specifically, low positive RF or anti-CCP account for 2 points and high positive RF or anti-CCP for 3 points, which correspond to 1/3 and half, respectively, of the minimum score required for RA classification^2^. These changes have attained the goal of increased sensitivity for patients in the early phases of RA at the cost of lower specificity^8, 9^. However, there is still room for improvement, both in sensitivity and specificity^10, 11^. It is, therefore, of interest to consider if new RA autoantibodies, the anti-carbamylated protein (anti-CarP) antibodies, could contribute to the early classification of patients with RA and no study has yet done this specific analysis.

The anti-CarP antibodies target proteins that have experienced a post-translational modification, the carbamylation of lysine residues into homocitrulline^12^. However, we do not know yet the proteins that are targeted in patients. In their place, the antibodies are assayed against *in vitro* carbamylated fetal calf serum (FCS)^12–15^. These assays have shown that the anti-CarP antibodies are specific of RA, and precede clinical onset of the disease by many years (similar to the anti-CCP antibodies)^14–16^. In addition, the anti-CarP antibodies are associated with bone erosions, disease activity, disability and mortality in RA with independence of the anti-CCP antibodies^12, 16–19^. All these characteristics indicate that the anti-CarP antibodies could add to the classification of RA patients. In previous studies, one with arthralgia patients^20^, the other with early arthritis (EA) patients^21^, the anti-CarP antibodies were associated with the RA outcome with independence of other autoantibodies. In addition, the anti-CarP antibodies seemed to contribute to classification in, at least, some subsets of patients. However, their value is still unclear because the extent of this contribution was modest and not quantified.

Here, we aimed to obtain a more defined conclusion on the value of anti-CarP antibodies for the classification of EA patients. This should be possible by a combination of two factors: the use as classification outcome the 1987 criteria to avoid bias in the assessment of the anti-CarP antibodies relative to RF and the anti-CCP antibodies^7^; and adoption of analysis focused in the improvement of current classification criteria (considering RF or anti-CCP antibodies)^2^.

## Material and Methods

### Patients and samples

Patients were recruited in two Spanish EA prospective clinics (EAC), at Hospital Universitario La Paz (from January 1993 to December 2013)^22^, and from PEARL (Princesa Early Arthritis Register Longitudinal) study at Hospital Universitario La Princesa (from July 2001 to December 2014)^23^, both in Madrid. The patients were selected for the EA cohort if they had showed 2 or more swollen joints for less than a year, and if they were naïve for Disease Modifying Anti-Rheumatic Drugs (DMARD). In addition, EA patients were selected for this study if they have completed 2 years of follow-up and if a serum sample from the baseline visit was available (n = 525 from La Paz and 537 from PEARL; Supplementary Table S1). These patients were classified at the end of the 2-year follow-up according to the 1987 American College of Rheumatology (ACR) classification criteria for RA^7^. Also, 208 healthy controls were recruited at Hospital Clinico Universitario of Santiago to set the anti-CarP antibody threshold. They were selected as subjects with good health coming to the hospital for elective ambulatory minor surgery providing consent to participate. About half (49%) were women and their median age was 69 years (IQR = 63-75). The EAC and the sample collections were approved by the La Paz University Hospital Ethics Committee and the Ethics Committee for Clinical Research of Hospital Universitario La Princesa (Ref. PI-518). The study was approved by the Autonomous Research Ethics Committee of Galicia (Ref. 2014/387). All participating subjects gave their written informed consent and all protocols and methods were conducted according with the relevant guidelines (Declaration of Helsinki and the Belmont Report) and regulations (Spanish Law 14/2007 of Biomedical Research).

### Generation of the carbamylated proteins and determination of autoantibodies

We used FCS (F-7524, Sigma-Aldrich) as source of proteins for testing anti-CarP reactivity. *In vitro* carbamylation of proteins from FCS was performed by incubating 4 mg/mL FCS with 1 M KCNO at 37 °C 15 h as previously described^12, 17^. The efficiency and percentage of carbamylation was corroborated by HPLC as previously done^17^. Anti-CarP IgG antibodies were quantified by ELISA as described in detail^12, 17^. All samples were assayed in separate plates coated with FCS and with carbamylated FCS and reactivity to native FCS was subtracted from the reactivity to carbamylated FCS. A standard curve made with serial dilutions from a pool of positive sera was used to measure antibody titers in arbitrary units. The cut-off for positivity was set at 98% specificity level obtained in the 208 healthy controls. The antibody titers in the controls did not differ between women and men (t-test p = 0.5), or in function of age (Spearman correlation p = 0.5). The other autoantibodies were determined as part of the routine care of the patients. IgM-RF was determined by nephelometry, whereas anti-CCP antibodies were determined by ELISA. The anti-CCP antibodies were determined as anti-CCP2 with the Euro-Diagnostica Immunoscan RA (positive >50 U/ml) in all the patients of the La Paz EAC and in the patients of PEARL until October 2010. Thereafter, the PEARL samples were analyzed as anti-CCP3 with the QUANTA Lite CCP3 IgG and IgA assay of Inova Diagnostics (positive >40 U/ml).

### Statistical analysis

Dichotomous patient features were compared with 2 × 2 contingency tables, whereas continuous variables were compared with t-test for independent samples or the U of Mann-Whitney test. Goodman and Kruskal’s gamma coefficient (γ) was used to measure concordance positivity between antibodies (ranging from +1 = perfect concordance to −1 = complete discordance). Parameters measuring the performance of the different antibodies in classification included sensitivity, specificity, positive predictive value (PPV), negative predictive value (NPV), positive likelihood ratio (LR+) and negative likelihood ratio (LR−). In addition, the area under (AUC) the receiver operating characteristic (ROC) curves was obtained using SPSS version 15.0 (Chicago, USA). Logistic regression was used to identify association between the antibodies and the classification as RA accounting for the anti-CCP and RF status and including age, sex and the EAC as covariates. All the previous analyses considered only two status for each antibody, positive or negative. The next analyses considered three levels following the protocol used during development of the 2010 ACR/European League Against Rheumatism (EULAR) classification criteria^24^. These three levels were: negative, positive below the median, and positive over the median. The median was calculated with the titers of the positives in each of the two EAC separately. In addition, the median of anti-CCP antibodies of the PEARL were calculated specifically for each of the two assays employed. The classification potential of the three antibody levels was compared between anti-CarP antibodies and the combined “RF or anti-CCP” by means of logistic regression (with age, sex and the EAC as covariates). In addition, classification trees of the C&RT style^25^, which perform exhaustive search for univariate splits, were applied to the three level status of anti-CarP, RF and anti-CCP antibodies in an ordinal scale. The objective was exploratory, not of establishing new classification algorithms. The Gini measure was used for assessing the tree’s goodness of fit, whereas the fraction of patients remaining to classify was used to limit the length of the tree modifying it until the anti-CarP antibodies appeared for the first time. Statistical analysis was performed with Statistica version 7.0 (StatSoft, Tulsa, OK) except for the ROC analysis, as mentioned.

### Data availability

The datasets generated during the current study are available from the corresponding author on reasonable request.

## Results

### Anti-CarP antibodies in the EA patients

There were 1062 patients with EA fulfilling the requirements for our study, completed 2 year follow-up and available baseline serum (Table 1). Patients from the two EAC showed some differences, including that patients from the La Paz EAC were younger, showed earlier arthritis, and were classified less often as RA at the end of follow-up than the patients from PEARL (Supplementary Table S1).

**Table 1:** Clinical and serological features of the EA patients.

Feature	Patients (n = 1062)	RA (n = 530)	non-RA (n = 532)	*P*
Women (%)	818/1062 (77.0)	420/530 (79.2)	398/532 (74.8)	0.09
Years of age at onset, median (IQR)	52 (40–65)	54 (43–67)	49 (37–64)	2.5 × 10^−5^
Weeks since onset, median (IQR)	16 (8–28)	20 (11–3)	12 (6–24)	6.3 × 10^−4^
Smoker (%)	444/984 (45.1)	234/509 (46.0)	210/475 (44.2)	0.6
RF positive (%)	444/1062 (41.8)	358/530 (67.5)	86/532 (16.2)	6.4 × 10^−65^
anti-CCP positive (%)	395/1046 (37.8)	349/526 (66.3)	46/520 (8.8)	2.7 × 10^−82^
anti-CarP positive (%)	291/1062 (27.4)	222/530 (41.9)	69/532 (13.0)	6.7 × 10^−24^
DAS28 (median, IQR)	4.5 (3.4–5.8)	5.2 (3.9–6.3)	3.9 (2.9–4.9)	7.0 × 10^−34^
Erosions (%)	93/883 (10.5)	77/529 (14.6)	16/354 (4.5)	6.5 × 10^−6^

Characteristics of the EA patients as a whole and stratified as RA and non-RA at the end of the two-year follow-up. *P* values correspond to the comparison of the two strata with t-student or chi-squared tests. IQR = interquartile range.

**Figure 1:**
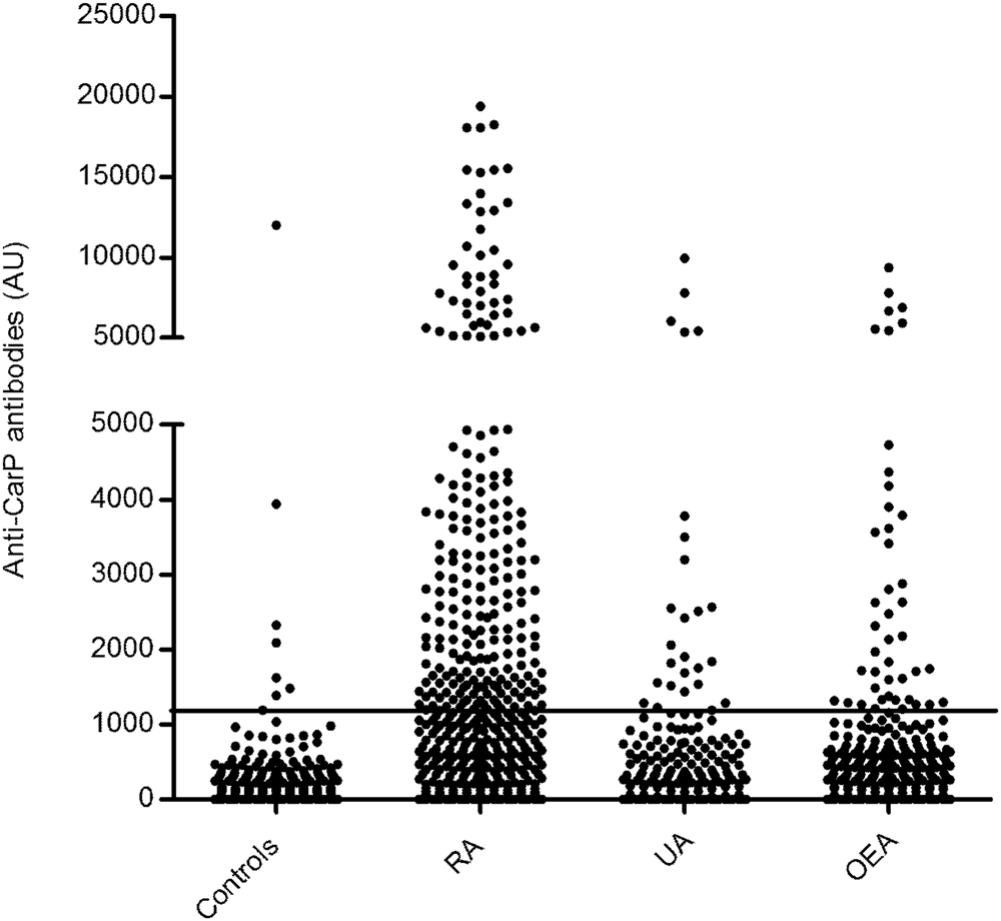
Titers of anti-CarP antibodies in the EA patients and healthy controls. The EA patients were stratified according with their classification at the end of the two year follow-up in RA, undifferentiated arthritis (UA) and other EA (OEA). Each dot represents the anti-CarP titer of a subject. The horizontal line is the threshold for positivity. The Y axis break separates two sections with different scaling without solution of continuity.

By the end of follow-up, about half of the EA patients (49.9%) fulfilled the criteria for RA. The remaining patients showed a variety of diseases, including undifferentiated arthritis (UA) as the most common (20%), and other less common diseases as spondyloarthritis, Sjögren syndrome, systemic lupus erythematosus, psoriatic arthritis, inflammatory bowel disease …, which jointly added to 30.1% of the total and were considered as the other EA group (OEA). The RA patients and the non-RA patients were different at some baseline features (Table 1), including that the RA patients were older, showed longer duration of symptoms, and were more often smokers than the non-RA patients. Frequencies of the RA antibodies were also disparate in RA and non-RA patients, with the anti-CCP antibodies as the most different, followed by RF and the anti-CarP antibodies in this order (Table 1). These last antibodies were positive in 27. 4% of the EA patients, predominantly in patients with RA (41.9%), but with a significant presence in the non-RA patients (13.0%), and no differences between UA (13.8%) and OEA (11.8%) patients (Fig. 1). It is worth to note that the anti-CarP titers among the positives were not different between the three groups of patients (median titers of the positives in RA, UA and OEA = 2780, 2060 and 2160, respectively).

The anti-CarP antibodies were concordant with the other two antibody types (γ = 0.70 with either anti-CCP or RF) in the RA patients, but less concordant than RF and anti-CCP between them (γ = 0.89). In the non-RA patients, the three antibodies were less concordant, and again the anti-CarP antibodies showed a lower concordance with RF (γ = 0.52) and with anti-CCP (γ = 0.39) than anti-CCP with RF (γ = 0.74). The correlations among antibody positive patients did not prevent an independent association of the anti-CarP antibodies with RA, as shown by logistic regression analysis accounting for anti-CCP and RF (OR = 1.7, 95% CI = 1.1–2.6, p = 0.009). However, this multivariate analysis including the three antibodies showed also that the association of anti-CarP antibodies with RA was notably weaker than the association of anti-CCP antibodies (OR = 11.3, 95% CI = 7.4–17.2, p = 3.5 × 10^−29^) and even than that of RF (OR = 2.9, 95% CI = 2.0–4.3, p = 2.9 × 10^−8^).

### Diagnostic characteristics of the anti-CarP antibodies in EA patients

We have analyzed in depth sensitivity and specificity, which are the most basic and understandable parameters assessing diagnostic performance (Table 2). As already shown above, the anti-CarP antibodies were the less sensitive of the three antibodies. However, they showed high specificity, which was almost as high as the observed with the anti-CCP antibodies. The sensitivity of anti-CarP antibodies was much decreased, 17.5%, in the subset of patients lacking anti-CCP (Table 2), where it was inferior to the sensitivity of RF. It was further decreased, to 9.4%, in the seronegative RA patients, those lacking both anti-CCP antibodies and RF. In contrast, specificity remained high in these two subsets of patients. These results could indicate a potential, but modest, value of anti-CarP antibodies to classify patients lacking other antibodies.

**Table 2:** Specificity and sensitivity for RA of the antibodies and their combinations.

	anti-CarP	anti-CCP	RF	anti-CCP or RF	anti-CCP or RF or anti-CarP	(anti-CCP or RF) & anti-CarP
**Sensitivity**						
all	41.9	66.3	67.5	75.9	78.1	39.7
anti-CCP^−^	17.5	na	28.2	28.2^a^	35.0	10.7
anti-CCP^−^ & RF^−^	9.4	na	na	na	9.4^a^	na
**Specificity**						
all	87.0	91.2	83.8	79.4	71.3	94.8
anti-CCP^−^	87.8	na	87.1	87.1^a^	78.3	96.6
anti-CCP^−^ & RF^−^	89.8	na	na	na	89.8^a^	na

Antibodies were combined with either the or operator of the and (&) operator. All in the row headings refers to all RA patients, the other rows refer to the indicated subset of RA patients. ^a^Values already presented in the table to the right, but duplicated to facilitate comparison. na = not applicable.

The most relevant contribution of the anti-CarP antibodies will be to provide increased sensitivity over the combination “anti-CCP or RF positivity” used in the current 2010 criteria. This combination resulted in an increase of 8.4% in sensitivity at the cost of a loss of 4.4% of specificity taken as reference the RF status (difference between “RF” and “anti-CCP or RF” columns in Table 2). Alternatively, the “anti-CCP or RF positivity” combination increased 9.6% sensitivity at a cost of 11.8% in specificity if the anti-CCP status was taken as reference instead. In contrast, the inclusion of the anti-CarP antibodies into the combined positivity criterion (“anti-CCP or RF or anti-CarP”) resulted only in an additional increase of 2.2% in sensitivity at the cost of a decrease in 8.1% in specificity (difference between the “anti-CCP or RF” and the “anti-CCP or RF or anti-CarP” columns in Table 2). A slightly more notable impact was observed in the subsets of patients lacking anti-CCP, or lacking both anti-CCP and RF (Table 2), with gains in sensitivity of 6.8% and 9.4%, respectively, and preserved specificity (Table 2).

Classifications based in the AND operator between antibodies increased specificity, but with a notable decline of sensitivity (as exemplified in the leftmost column of Table 2), which is the parameter that needs to improve.

PPV, NPV, LR+, LR− and AUC of the ROC were calculated to inform about the performance of the antibodies in the classification of EA patients (Table 3). The most relevant are the values pertaining to combinations of antibodies. In one side, NPV and LR−, which assess the exclusion of RA in patients lacking the antibodies, did not decrease by the inclusion of anti-CarP antibodies in the combination (i.e. for all patients NPV = 75.6% *vs* 75.3% in the combination without and with anti-CarP, respectively; and LR− = 0.3 both for the combination without and with anti-CarP; Table 3 for subgroups of patients). In contrast, PPV and LR+, which evaluate the classification as RA of the antibody positive patients, were decreased by the incorporation of the anti-CarP antibodies (i.e. for all patients PPV = 78.9% *vs* 73.4% in the combination without and with anti-CarP, respectively; and LR+= 3.7 *vs* 2.7 in the combination without and with anti-CarP, respectively; Table 3 for subgroups of patients). Finally, the AUC showed no improvement in the combinations incorporating anti-CarP antibodies over the combinations without them (i.e. for all patients AUC = 0.77 *vs* 0.74 in the combination without and with anti-CarP, respectively; Table 3 for subgroups of patients).

**Table 3:** Additional parameters assessing the diagnostic value of the antibodies and their combination.

	anti-CarP	anti-CCP	RF	anti-CCP or RF	anti-CCP or RF or anti-CarP
**PPV**					
all	76.3	88.4	80.6	78.9	73.4
anti-CCP^−^	34.8	na	45.0	45.0^a^	37.6
anti-CCP^−^ & RF^−^	22.2	na	na	na	22.2^a^
**NPV**					
all	60.1	72.8	72.2	76.5	76.3
anti-CCP^−^	74	na	76.5	76.5^a^	76.3
anti-CCP^−^ & RF^−^	76.3	na	na	na	76.3^a^
**LR+**					
all	3.2	7.5	4.2	3.7	2.7
anti-CCP^−^	1.4	na	2.2	2.2^a^	1.6
anti-CCP^−^ & RF^−^	0.9	na	na	na	0.9^a^
**LR−**					
all	0.7	0.4	0.4	0.3	0.3
anti-CCP^−^	0.9	na	0.8	0.8^a^	0.8
anti-CCP^−^ & RF^−^	1.0	na	na	na	1.0^a^
**AUC**					
all	0.65	0.79	0.76	0.77	0.74
anti-CCP^−^	0.53	na	0.58	0.58^a^	0.57
anti-CCP^−^ & RF^−^	0.49	na	na	na	0.49^a^

All refers to all RA patients, the other rows refer to the indicated subset of RA patients. ^a^Values already presented in the table to the right, but duplicated to facilitate comparison. PPV = positive predictive value, NPV = negative predictive value, LR + = likelihood ratio of a positive finding, LR− = likelihood ratio of a negative finding and AUC = area under the Receiver Operating Curve. na = not applicable.

### Value of the anti-CarP antibodies in the context of the RA classification criteria

In the logistic regression analysis that followed the methods of the 2010 RA classification criteria^24^, we have found a very strong association of high positive titers of anti-CarP antibodies (OR = 7.1) with RA (Table 4). However, this association was much lower than the obtained with high titers of “anti-CCP or RF” (OR = 36.4). The low titer anti-CarP positives showed also strong association with RA (OR = 3.9) that was nominally, but not significantly lower than the found with high titers of anti-CarP antibodies. In addition, the association of the subgroup of low anti-CarP titers was not significantly different from the found with low titers of “anti-CCP or RF” (OR = 5.1). Therefore, high and low titers of anti-CarP could be considered together and with a similar weight as the given to low titers of the “anti-CCP or RF” criterion.

**Table 4:** Logistic regression analysis of the strength of association of different antibody levels.

Antibody	Comparison	B	*P*	OR (95% CI)
anti-CCP or RF	low *vs* negative	1.6	1.3 × 10^−18^	5.1 (3.5–7.3)
anti-CCP or RF	high *vs* negative	3.6	3.2 × 10^−30^	36.4 (19.6–67.3)
anti-CarP	low *vs* negative	1.4	2.4 × 10^−11^	3.9 (2.6–5.8)
anti-CarP	high *vs* negative	2.0	1.1 × 10^−17^	7.1 (4.5–11.1)

Each antibody category was analyzed separately with age, sex and EAC as covariates. Low and high categories were defined in relation with the median of the positives.

**Figure 2:**
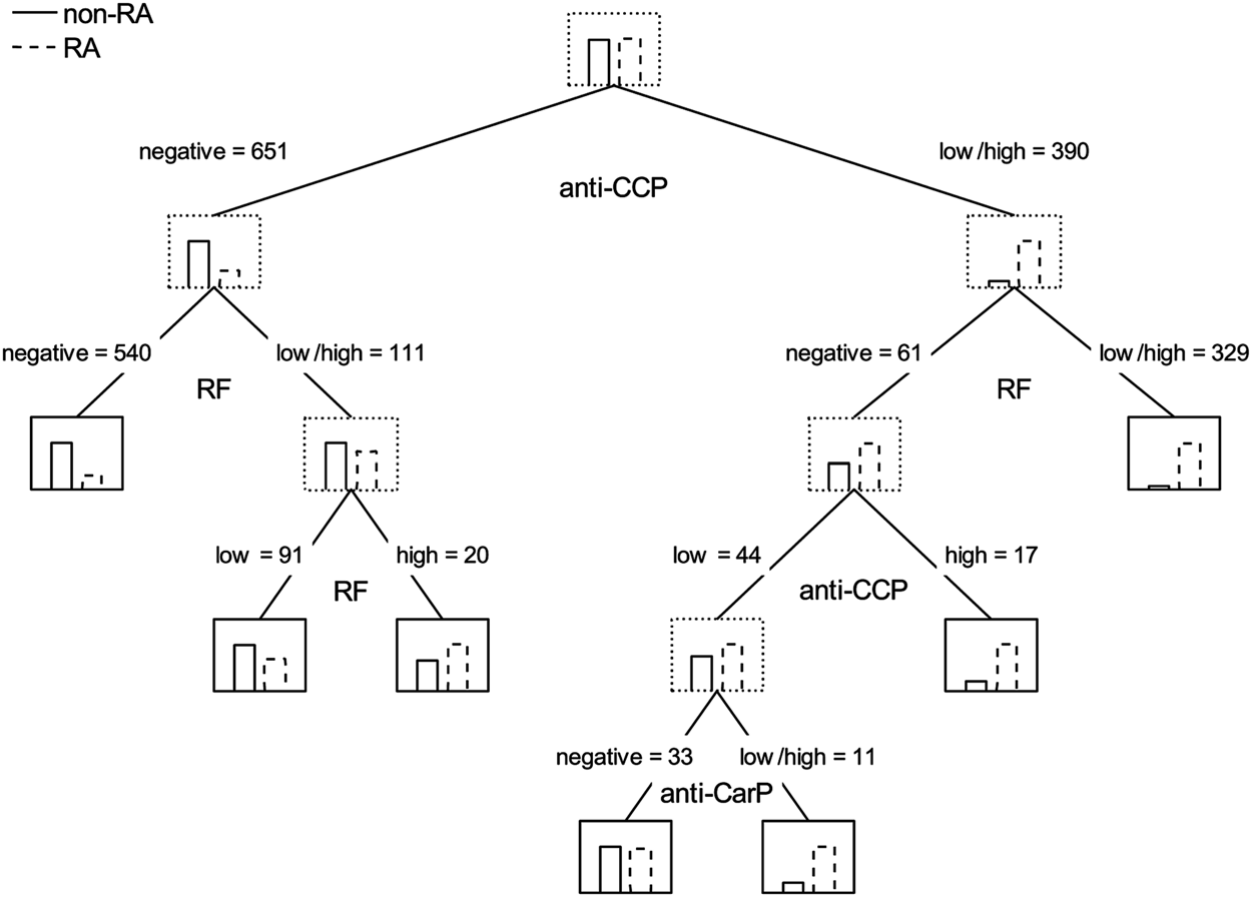
Smallest classification tree of the EA patients according with their antibody status in which the anti-CarP antibodies appear. This tree is not a valid classification tool, it is only an exploratory analysis of the role of anti-CarP antibodies. Each of the antibodies was considered as negative, low positive and high positive in an ordinal scale. The tree was allowed to grow until more than 15 RA and 15 non-RA patients remained to classify in any of the two groups, which was the smallest tree showing a split based on anti-CarP antibodies. Node lines are dotted, except for terminal nodes that show continuous lines. Nodes include histograms with the frequencies of the RA (discontinuous lined bar) and non-RA (continuous lined bar) patients at that level of the tree. The antibody used for each split is indicated under parent nodes. Over each child node appears the rule to reach it and the number of patients arriving to it.

Classification trees following the same premises showed a dominant role of the anti-CCP antibodies, which were used in the first split to classify as RA all anti-CCP positive patients, followed by additional splits according to the presence of RF (Fig. 2). The first tree in which the anti-CarP antibodies appeared was large, including 6 splits and 7 terminal nodes (Fig. 2). In it, the contribution of the anti-CarP antibodies did not increase sensitivity as needed, but specificity by splitting patients that were low positive for anti-CCP and negative for RF.

## Discussion

We are the first to attempt defining the contribution of anti-CarP antibodies to the classification of RA in EA patients. This contribution was evaluated in relation with clinical needs and inspired by the current classification criteria^1–5, 24^. The resulting characteristics of the contribution can be described in four points. First, we have confirmed that the anti-CarP antibodies are independently associated with RA among EA patients. We have also found that the strength of this association is modest when accounting for the other RA autoantibodies. Second, the inclusion of anti-CarP antibodies in a classification rule jointly with anti-CCP and RF was only advantageous with the OR operator. In addition, no significant improvement in specificity was obtained with complex or alternative classification algorithms. Third, the “anti-CCP or RF or anti-CarP” rule produced an increase in sensitivity of 2.2% at the cost of a decrease in specificity of 8.1% over the existing “anti-CCP or RF” criterion. This will mean roughly to misclassify 4 non-RA patients as RA for each new RA patient that is detected. Fourth, any eventual rule including the anti-CarP antibodies will not need to distinguish between high and low titers, as we did not find significant differences between them. These points will be useful to establish the anti-CarP antibodies role in future modifications of the RA classification criteria. However, our data already indicate they will not add a significant improvement. These results contrast with the imprecise suggestion derived from previous studies proposing the anti-CarP antibodies could be useful for RA classification.

In effect, our results were similar to the published before in some respects and different in others. The similar aspects include: independent but modest association of the anti-CarP antibodies with RA, their modest sensitivity in anti-CCP negative patients, and their high specificity for RA^14–16, 20, 21^. This consistency of results is manifest also when the parameters measuring the diagnostic performance of the anti-CarP antibodies are directly compared one by one with the previously reported for EA patients^21^ (Supplementary Table S2). However, no precedent is available for the focused information we have additionally obtained. That is, there is no precedent of a cost-benefit ratio showing no significant improvement by including anti-CarP antibodies, or of the absence of alternative classification rules with better performance.

Our study also differed from the previous reports^20, 21^ in the RA classification criteria that were used. Here, they were from 1987^7^, whereas in previous studies, they were from 2010^2^. The first are less sensitive in the first months since symptoms onset, whereas the second are less specific^8, 9, 11^. In addition, the 2010 criteria seem to introduce changes in the phenotype of patients, towards milder disease and an increased ratio of seropositive to seronegative patients^10, 11^. In the three studies, the criteria were applied after some follow-up, which in our study was of two years. This was one of the justifications of our choice, because after this follow-up the features included in the 1987 criteria are more sensitive than if they were applied at the first visit. In our view, this approach could obtain a good balance of specificity and sensitivity, acknowledging that no perfect tool for classification is yet available^10, 11^. However, the main reason motivating our election of the 1987 criteria was that they give less weight to the antibodies than the 2010 criteria. In effect, it is widely acknowledged that an important fraction of the differences between the two RA classification criteria is due to the higher contribution of the antibodies to the most recent score^8, 9^. This important contribution of the antibodies to the 2010 criteria hampers the accurate assessment of the contribution of the anti-CarP antibodies relative to the other antibodies.

The benefit achievable with the incorporation of anti-CarP antibodies to RA criteria appears significantly smaller than the obtained by the “anti-CCP or RF” combination and it will incur higher costs in specificity. According to our results, the benefit of including RF was the largest, increasing sensitivity with respect to using only the anti-CCP status by 9.6%, at the lowest cost-benefit ratio, roughly 1:1. In contrast, the inclusion of anti-CarP antibodies produced an increase in sensitivity a fourth of that at a cost-benefit ratio four times larger. These numbers mean that incorporation of anti-CarP antibodies to the criteria is dubious unless very safe and effective treatments become available or other kind of information improves the value of the anti-CarP antibodies. This analysis could be relevant also for other new RA specific antibodies not yet analyzed for their value in classification^26–28^. If these new RA antibodies correlate with the already known, we should expect from our results that they will provide small improvements in sensitivity at some cost in specificity and that combinations with the OR operator will be the only viable. In contrast, antibodies showing a markedly different distribution, with many positive patients among the seronegative and showing enough specificity, will be more advantageous^28^.

It is important to do no draw conclusions about the clinical value of the anti-CarP antibodies that go beyond our results and analysis. Even if our conclusion is confirmed, the anti-CarP antibodies could still have a significant impact on the management of RA patients. They have shown independent association with joint erosions in multiple studies^12, 16, 17^. In addition, they are associated with a more severe disease according to disease activity, long term disability and mortality in some patient cohorts^18, 19, 29^, although not in others^10, 29^; and we cannot discard that future refinements in the test to determine the anti-CarP antibodies, as the use of relevant human antigens, could increase their clinical utility.

There are other limitations to our conclusion, including the nature of the two hospitals and of the health system where the two early arthritis clinics are established. They are academic medical centers in a large town and part of a public health system with universal coverage. These circumstance will make the results not applicable to other health systems or to primary care. In addition, the anti-CarP antibodies were determined with carbamylated FCS proteins and these results are different to the obtained with other sources of antigen as we have already found in preliminary experiments (unpublished). Other limitation is the possible error in assessment of the value of the anti-CarP antibodies introduced by the presence of RF in the 1987 RA classification criteria^7^. The weight of RF in that criteria is lower than in the 2010 criteria, but still it could bias results. We were only able to show that this effect would not be large in an indirect analysis (Supplementary Table S3).

In conclusion, the value of including the anti-CarP antibodies to criteria for classification of RA in EA patients has been defined and quantified in detail for the first time. The improvement is likely to be modest overall, with an increase of 2.2% in sensitivity, but with a higher impact in the subsets lacking other RA autoantibodies. This benefit should be balanced with the cost it will incur, because the loss of specificity roughly means that for each new RA patient identified 4 non-RA patients will be misclassified. Therefore, the place in RA classification criteria of these antibodies is not significant, at least, in the current context of treatments and of tools for patient classification.

## Electronic supplementary material

Supplementary tables
