# Supplementary material for: Value of Measuring Anti-Carbamylated Protein Antibodies for Classification on Early Arthritis Patients
Source: Sci Rep. 2017 Sep 20;7:12023. doi: 10.1038/s41598-017-09657-5 (PMC5607236; doi:10.1038/s41598-017-09657-5)
Supplement: Supplementary file 1 — Supplementary tables [file 41598_2017_9657_MOESM1_ESM.doc]

**Supplementary material**

**Value of Measuring Anti-Carbamylated Protein Antibodies for Classification on Early Arthritis Patients**

**Cristina Regueiro^1^, Laura Nuño^2^, Ana M. Ortiz^3^, Diana Peiteado^2^, Alejandro Villalba^2^, Dora Pascual-Salcedo^2^, Ana Martínez-Feito^4^, Isidoro González-Alvaro^3^, Alejandro Balsa^2^, Antonio González^1,*^**

1.- Experimental and Observational Rheumatology. Instituto de Investigacion Sanitaria - Hospital Clínico Universitario de Santiago, Santiago de Compostela, Spain

2.- Rheumatology Department. Instituto de Investigación Hospital Universitario La Paz (IDIPAZ), Madrid, Spain

3.- Rheumatology Department. Instituto de Investigación del Hospital de La Princesa (IIS-IP), Madrid, Spain.

4.- Immuno-Rheumatology Department. Instituto de Investigación Hospital Universitario La Paz (IDIPAZ), Madrid, Spain*

*[agmartinezp@ser.es](mailto:agmartinezp@ser.es)

**Supplementary table S1. Comparison of the characteristics of patients from the two EAC.** Classification in RA, UA and OEA was done at the end of the two year follow-up. *P* values correspond to the comparison of the two strata with t-student or chi-squared tests. IQR= interquartile range

| Feature | Total (n=1062) | EAC La Paz (n=525) | EAC La Princesa (n=537) | *P* |
| --- | --- | --- | --- | --- |
| Women (%) | 818/1062 (77.0) | 389/525 (74.1) | 429/537 (79.9) | 0.025 |
| Years of age at onset, median (IQR) | 52 (40-65) | 50 (39-65) | 54 (42-66) | 0.015 |
| Weeks from symptom onset to first visit, median (IQR) | 16 (8-28) | 12 (6-22) | 21 (12-34) | < 0.001 |
| Smoker (%) | 444/984 (45.1) | 222/477 (46.5) | 222/507 (43.8) | 0.001 |
| RF positive (%) | 444/1062 (41.8) | 217/525 (41.3) | 227/537 (42.3) | 0.757 |
| anti-CCP positive (%) | 395/1046 (37.8) | 201/525 (38.3) | 194/521 (36.1) | 0.726 |
| anti-CarP positive (%) | 291/1062 (27.4) | 146/525 (27.8) | 145/537 (27.0) | 0.768 |
| RA (%) | 530/1062 (49.9) | 252/525 (48.0) | 278/537 (51.8) | 0.219 |
| UA (%) | 212/1062 (20.0) | 90/525 (17.1) | 122/537 (22.7) | 0.023 |
| OEA (%) | 320/1062 (30.1) | 183/525 (34.9) | 137/537 (25.5) | < 0.001 |

**Supplementary table S2.** **Diagnostic parameters of anti-CarP, anti-CPP and RF antibodies for RA in the current study compared with those in Leiden EAC.** PPV= positive predictive value, NPV= negative predictive value, LR+= positive likelihood ratio, LR- = negative likelihood ratio, AUC= area under the curve.

|  |  | EA Leiden (n=2086) | Current study (n=1062) |
| --- | --- | --- | --- |
| EAC | % anti-CarP positive | 26 | 27 |
|  | % RA | 47 | 50 |
|  |  |  |  |
| anti-CarP | sensitivity | 44 | 42 |
|  | specificity | 89 | 87 |
|  | sensitivity in anti-CCP^-^ | 12 | 18 |
|  | specificity in anti-CCP^-^ | 91 | 88 |
|  | PPV | 78 | 76 |
|  | NPV | 65 | 61 |
|  | LR + | 4.2 | 3.2 |
|  | LR - | 0.6 | 0.7 |
|  | AUC | 0.7 | 0.6 |
|  |  |  |  |
| anti-CCP | sensitivity | 54 | 66 |
|  | specificity | 96 | 91 |
|  | PPV | 94 | 88 |
|  | NPV | 64 | 73 |
|  | LR + | 12.9 | 7.5 |
|  | LR - | 0.5 | 0.4 |
|  | AUC | 0.5 | 0.8 |
|  |  |  |  |
| RF | sensitivity | 59 | 68 |
|  | specificity | 91 | 84 |
|  | PPV | 86 | 81 |
|  | NPV | 72 | 72 |
|  | LR + | 6.9 | 4.2 |
|  | LR - | 0.4 | 0.4 |
|  | AUC | - | 0.8 |

**Supplementary table S3: Comparison of the anti-CarP performance in RF negative patients with anti-CCP negative patients.** This comparison intends to find if the presence of RF in the 1987 RA classification criteria introduce a significant error in the assessment of the value of the anti-CarP antibodies for classification. The RF negative patients can not be affected by this possible bias, therefore we compared the anti-CarP test in this subgroup of patients with its performance in the anti-CCP negative patients. These latter were used as reference because the anti-CCP antibodies are not included in the 1987 criteria. No significant differences were found. This result could be interpreted as excluding important error. However, it should be noted that this comparison has less power than other analyses in the main text because it concerns a subgroup of patients and because the antibodies are correlated.

|  |  | | RF^-^ (n=618) | | Anti-CCP^-^ (n=651) | |
| --- | --- | --- | --- | --- | --- | --- |
| anti-CarP | sensitivity | | 17 | | 18 | |
|  | specificity | | 90 | | 88 | |
|  | PPV | | 39 | | 35 | |
|  | NPV | | 74 | | 74 | |
|  | LR + | | 1.6 | | 1.4 | |
|  | LR - | | 0.9 | | 0.9 | |
|  | |  | |  | |  |
